# Screening of *MITF* and *SOX10* Regulatory Regions in Waardenburg Syndrome Type 2

**DOI:** 10.1371/journal.pone.0041927

**Published:** 2012-07-27

**Authors:** Viviane Baral, Asma Chaoui, Yuli Watanabe, Michel Goossens, Tania Attie-Bitach, Sandrine Marlin, Veronique Pingault, Nadege Bondurand

**Affiliations:** 1 INSERM, U955, equipe11, Créteil, France; 2 Université Paris Est, Faculté de Médecine, Créteil, France; 3 AP-HP, Hôpital H.Mondor-A. Chenevier, Service de biochimie et génétique, Créteil, France; 4 INSERM U781, Université Paris Descartes, Hôpital Necker-Enfants Malades, Paris, France; 5 Service de Génétique, Centre de référence «Surdités génétiques», INSERM U587, Hôpital Armand Trousseau, APHP, Paris, France; Innsbruck Medical University, Austria

## Abstract

Waardenburg syndrome (WS) is a rare auditory-pigmentary disorder that exhibits varying combinations of sensorineural hearing loss and pigmentation defects. Four subtypes are clinically defined based on the presence or absence of additional symptoms. WS type 2 (WS2) can result from mutations within the *MITF* or *SOX10* genes; however, 70% of WS2 cases remain unexplained at the molecular level, suggesting that other genes might be involved and/or that mutations within the known genes escaped previous screenings. The recent identification of a deletion encompassing three of the *SOX10* regulatory elements in a patient presenting with another WS subtype, WS4, defined by its association with Hirschsprung disease, led us to search for deletions and point mutations within the *MITF* and *SOX10* regulatory elements in 28 yet unexplained WS2 cases. Two nucleotide variations were identified: one in close proximity to the *MITF* distal enhancer (MDE) and one within the U1 *SOX10* enhancer. Functional analyses argued against a pathogenic effect of these variations, suggesting that mutations within regulatory elements of WS genes are not a major cause of this neurocristopathy.

## Introduction

Waardenburg syndrome (WS) is characterised by the association of sensorineural hearing loss and pigmentation abnormalities, including depigmented patches of the skin and hair and vivid blue eyes or heterochromia iridis. Its prevalence is estimated to be 1 in 42,000 and it is responsible for 1–3% of all cases of congenital deafness [1], [2]. Other features, such as dystopia canthorum, musculoskeletal abnormalities of the limbs, and Hirschsprung disease, are found in a subset of patients and used for the clinical classification of this syndrome into four subtypes (WS1-4). At the molecular level, WS is genetically heterogeneous, with six genes known to be involved: *PAX3* (encoding the paired box 3 transcription factor), *EDN3* (endothelin-3), *EDNRB* (endothelin receptor type B), *SOX10* (Sry bOX10 transcription factor), *MITF* (microphthalmia-associated transcription factor), and *SNAI2* (snail homolog 2) (for review, see [1]). WS2, which is defined by the absence of additional features, results from mutations occurring with different frequencies within the last three of these genes, *SOX10*, *MITF*, and *SNAI2*. Heterozygous *MITF* mutations have been reported in about 15% of cases [1], [2], but homozygous deletions of the *SNAI2* gene, however, have been described in only two patients [3], arguing against a major involvement of this gene. Recently, we showed that another 15% of WS2 cases are due to heterozygous *SOX10* point mutations or deletions [1], [4], [5]. Some mutations are responsible for extended phenotypes, including peripheral and central neurological defects, and are referred to as PCW (Peripheral demyelinating neuropathy - Central dysmyelinating leucodystrophy - Waardenburg syndrome) [1], [6]. Overall, 70% of WS2 remain unexplained at the molecular level, suggesting that other genes might be involved and/or that mutations within the known genes escaped previous screenings. It was therefore tempting to speculate that alteration of the expression level or sites of *MITF* or *SOX10*, which are tightly regulated during development, can lead to WS2.

Mitf/MITF, which encodes a member of the Myc supergene family of basic helix loop helix zipper (bHLH-Zip) transcription factors, is known as the key transcription factor in melanocyte development (for review, see [7], [8], [9]). This gene contains nine alternative promoters, producing multiple isoforms differing in their amino termini but sharing exons 2–9. Of all the different *Mitf* promoter elements, the melanocyte specific one (MITF-M) has generated the most interest because of its tissue specificity and function [8], [9], [10]. Various signalling molecules and transcription factors regulate expression from the MITF-M promoter, including Wnt, MSH, PAX3, SOX10, LEF-1, OC2, CREB, BRN2, and FoxD3 [9], [10], [11], [12]. In humans, most of the responsive *MITF* promoter sequences lie within a region of 400 bp upstream of the MITF-M transcription initiation site. A distal regulatory region known as the MITF distal enhancer, or MDE, was characterised more recently [13]. This region of 298 bp, localised nearly 15 kb upstream of the human MITF-M transcription initiation site, is partially conserved in mouse and dog [13], [14]. It contains at least two functional SOX10 binding sites and enhances M promoter activity in melanoma cells. In mouse, the importance of this element is consistent with the coat colour defects observed in the Mitf^mi-red-eyed-white^ mutant, carrying a large deletion including this region [9].

The SOX10 transcription factor is an important pleiotropic regulator of neural crest development, regulating stem cell maintenance and cell lineage progression (for reviews, see [15], [16], [17]). Its function is well described, and recent studies shed light onto the complex regulation of its expression [18], [19], [20], [21], [22], [23]. *In silico* analyses led to the identification of several enhancers of *SOX10*. We and others identified several of these regulatory elements, five upstream (U1–5) and one downstream (D6+7) of the human *SOX10* gene ([22], [23], [24]). The functional relevance of these elements was confirmed in different cell lines and in zebrafish, chicken and mouse models, where they drive expression in several neural crest derivatives [19], [22]. Two of them, U1 and U3, which are localised 55 kb and 33 kb upstream of the *SOX10* gene respectively, drive *SOX10*/*Sox10* expression during melanocyte development in particular, at least in zebrafish and in melanoma cells ([18], [19] and our unpublished results). These two sequences contain dimeric SOX consensus binding sites, which are essential for enhancer activity, as well as multiple binding sites for other factors known to play key roles in neural crest development [18], [19], [21].

Mutations within the *SOX10* gene are not only responsible for some WS2 cases but they also explain about 50% of WS4 cases, characterised by an association with Hirschsprung disease (HD, absence of enteric ganglia in the distal part of the intestine) [1], [25]. Recently, we described the first characterisation of a large deletion encompassing several *SOX10* enhancers in a patient presenting with WS4 [24]. Taken together with previous results, this demonstrated that the disruption of highly conserved non-coding elements located within or at a long distance from the coding sequences of key genes can result in several neurocristopathies, particularly WS and HD ([24], [26]). This led us to search for deletions and point mutations within the *MITF* and *SOX10* regulatory elements in unexplained WS2 cases.

## Materials and Methods

### Patients

A total of 28 WS2 patients previously found to be negative for point mutations or deletions within the *MITF* and *SOX10* genes were investigated. *SNAI2* screening revealed an absence of anomalies in the two patients presenting with MDE and U1 variations. Genomic DNA was extracted from peripheral blood leukocytes using standard protocols. Written informed consent was obtained for all patients. The study has been validated by the ethical committee which waived requirement for a formal ethical approval in regards to the research performed.

### Molecular analysis

Semi-quantitative fluorescent multiplex PCR (QMF-PCR) was used to amplify five of the regulatory regions located 5′ of the *SOX10* gene (U1-5) and one (D6+7) located 3′ of the gene in one fluorescently labelled multiplex reaction with two external controls, following previously described protocols ([24]). The same strategy allowed us to screen a 220 bp region of the *MITF* promoter using the following primers: 5′-TTAGATGATGTCTCCTCCAA-3′ and 5′-AAATGTTGATATCAATTTTTCC-3′.

In parallel, PCR amplification and direct sequencing of the U1, U3, MDE, and *MITF* promoter regions was performed using the primers described in Table 1. Thermo Scientific high fidelity DNA polymerase (Fermentas) was used for PCR amplification, with genomic DNA and 5% DMSO. The reaction started with an initial denaturation of 5 min at 95°C, followed by 35 cycles at 95°C for 1 min, 62°C (U1 and U3), 55°C (MITF promoter) or 58°C (MDE) for 1 min, and 72°C for 2 min. Then, 2 µl of the purified PCR products were used for direct sequencing.

**Table 1:** Sequences of primers used for PCR and sequencing of U1 and U3 SOX10 enhancers regions as well as *MDE* and promoter regions of *MITF* sequences.

	Primer sequence (5′→3′)		
Primer	Forward	Reverse	PCR size (bp)
***SOX10***			
U1 PCR	CCAGCCGCCCCCTACGACTGCCC	GCACAGGATGGGACGGGTTGAG	476
U1 SEQ	CCAGCCGCCCCCTACGACTGCCC	GTCGTCCAGGCGTTGAGTGT	
U3 PCR	CTCAGGAGGGCTGGAGAGTGGTG	GGGGCATCAGCGAATCTGTTTTG	902
U3 SEQ	TGCCAGGCAGCAGAGGCTGG	AGCAGAGCAAGGGCCTGGTG	
	TTCCAACATGTCATTACAGT	CGACGTTGACATTGTTCCCA	
	TGGGAACAATGTCAACGTCG		
***MITF*** ** promoter**			
PCR and SEQ	GCCCGGTCTTCCTGATGTGAGGTCA	GACTTATCCCTCCCTCTACTTTCTA	636
SEQ	TGATCTGACAGTGAGTTTGA	AGGCCAATTCACTATTCATC	
***MITF*** **-MDE**			
PCR	CCTGGGTTCAGGTGATTCTCCTG	AGCCCCTCAAGCCAGCAACGGG	652
SEQ	CAGGCATATGCCACCACACC	CGGAGAAAGTCAATATGGACATTTGTTC	

Expected PCR product size are reported. SEQ indicates the primers used for sequencing. pb: base pais.

Upon variation identification, the dbSNP (http://www.ncbi.nlm.nih.gov/snp) and 1000 genomes project (http://browser.1000genomes.org) databases were used to search for previously identified polymorphisms. In parallel, 50 controls (100 chromosomes) of matched geographical origins were confirmed negative for the variations identified in patients. The genomic location of the variations was given according to the international nomenclature based on the human chromosome 3 (NC_000003.11) and chromosome 22 (NC_000022.10) reference sequences. Analysis with the TFSEARCH program (Searching Transcription Factor Binding Sites, http://www.rwcp.or.jp/papia/) was used to seek putative transcription factor binding sites and their alteration upon variation identification.

### Plasmid constructs, cell culture, transfection, and reporter assays

The MDE reporter construct (previously called pGL3-cis1) was kindly provided by Pr. Shigeki Shibahara [13]. The identified variation was inserted by site-directed mutagenesis using the QuikChange Site-Directed Mutagenesis Kit (Stratagene). The U1 enhancer region was amplified by PCR using control and patient DNA and the primers 5′- GAGCTCCCAGCCGCCCCCTACGACTGCCC-3′ and 5′-CTCGAGGCACAGGATGGGACGGGTTGAG-3′, containing SacI and XhoI restriction sites, respectively. After double digestion, the PCR products were cloned into the pTAL-luc vector (Clontech). The FoxD3 cDNA was amplified using the primers 5′-GGCACTCAAACCCTCTTCCCCTGAGCTCCG -3′ and 5′-GCAGCCTGGAGGTGCATTTGTTGCT -3′, and cloned into a TOPO-V5 expression vector (Invitrogen).

HeLa and SKMel5 cells (ATCC) were grown in Dulbecco's modified Eagle's medium (DMEM) supplemented with 10% foetal calf serum and transfected using Lipofectamine PLUS reagents (Invitrogen). Approximately 110,000 cells were plated on 12-well plates and transfected 1 day later with 0.5 µg of reporter plasmid and the FoxD3 expression plasmid. Twenty-four hours post-transfection, cells were washed twice with PBS and lysed, and the extracts were assayed for luciferase activity using the Luciferase Assay System (Promega) as previously described [4], [5], [27].

## Results

### Analysis of *SOX10* regulatory sequences

**Figure 1:**
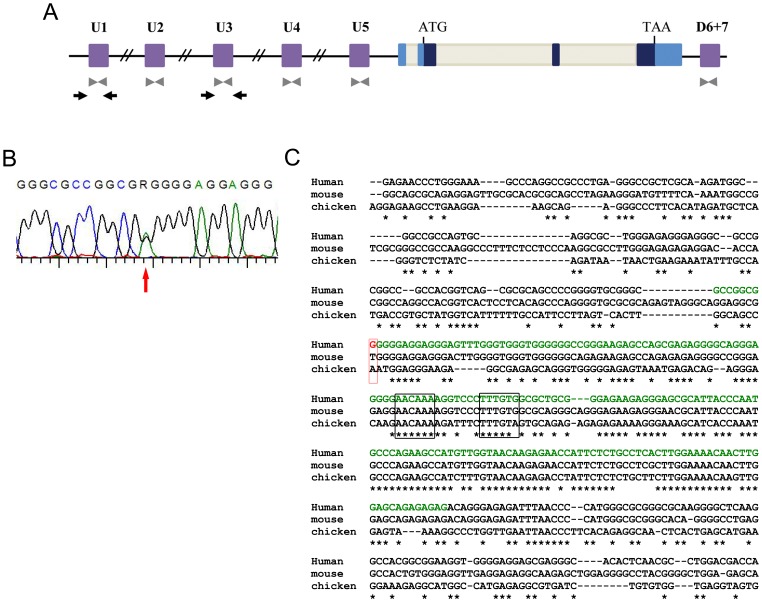
Variations identified in SOX10 regulatory regions. (A) Schematic view of the *SOX10* gene (start and stop codon are indicated) and putative enhancer regions located 100 kb around the human *SOX10* locus. SOX10-coding exons are in dark blue, noncoding exons in blue, intronic regions in grey and putative enhancers in purple. Grey arrowheads indicate the position of QMF-PCR primers. Black arrows indicate the position of primers used for PCR sequencing for screening. (B) Electropherogram showing the heterozygous variation identified. (C) Alignment of the nucleotide sequences of the human U1 region of *SOX10* (GenBank accession number NT_011520.12) and its corresponding *Mus musculus* (NT_039621.7) and *Gallus gallus* (NW_001471513.1) homologous regions. The asterisks indicate the nucleotides conserved between murine, chicken, and human sequences. The two previously described putative SOX10 binding sites are indicated by black open boxes. Nucleotides included in the region identified by DCODE analysis or previously published [22] are indicated in green. The location of the identified variation is indicated by a red open box.

Based on the recent identification of a WS4 patient presenting with a large deletion encompassing three SOX10 enhancers, we first screened for deletions of SOX enhancers using the previously described QMF-PCR strategy ([24]). Analysis of the U1–5 and D6+7 regions (Fig. 1A, grey arrowheads indicate the position of the primers) revealed an absence of deletions or detectable rearrangement within the 28 WS2 cases included in our study. The high enhancer activity of U1 and U3 sequences observed in melanoma cells ([19] and our unpublished results), along with their crucial function during zebrafish melanocyte development, led us to analyse these regions in more detail. We searched for point variations within these two regulatory elements (see Fig. 1A, black arrows, and Table 1 for primer sequences) by a direct PCR sequencing strategy. No variations were found within U3, but one was identified within U1: g.38434799C>T on chromosome 22 (G>A on the reverse sequence, Fig. 1B), which has not been reported in polymorphism databases. This nucleotide, which lies 5′ of the most conserved sequence, is not evolutionarily conserved (Fig. 1C). The patient was born of a healthy non consanguineous couple. He presented with a white frontal forelock and bilateral profound hearing loss revealed by neonatal hearing screening. Temporal bones CT scan showed no malformation and a cochlear implantation has been performed. His older sister presented with isolated, bilateral profound hearing loss diagnosed at the age of 6 months. No sign of skin, hair or irides depigmentation was observed. *GJB2* mutations screening was found negative. The parents and sister testing revealed the variation was inherited from the mother and was not carried by the sister.

**Figure 2:**
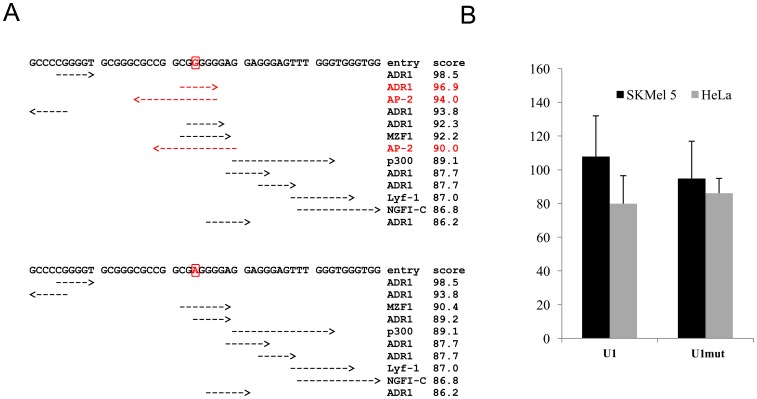
Functional analysis of the variation identified within U1. (A) TFSEARCH results obtained upon analysis of 50 bp around the variation. The top panel corresponds to the wild-type sequence, whereas the bottom panel corresponds to the mutated sequence. Variation and affected binding sites are indicated in red. (B) Functional consequences of the variation. Wild-type and mutated versions of the U1 sequences were cloned upstream of a minimal promoter driving basal luciferase expression and assayed for enhancer activity in SKMel5 and HeLa cells. Reporter gene activation is presented as fold-induction relative to the empty vector. Results represent the mean ± standard error of three to five different experiments, each performed in duplicate.

TFSEARCH analysis indicated that the concerned variation may alter putative ADR1 (alcohol dehydrogenase (ADH) II synthesis regulator) and/or AP-2 (activator protein-2) binding sites (Fig. 2A).

The effect of this variation on the ability of U1 to direct reporter gene expression was tested *in vitro*. To this end, wild-type or mutated versions of U1 were cloned upstream of a minimal promoter directing basal luciferase expression, and constructs were transfected into SKMel5 and HeLa cell lines, and their enhancer activity was tested 24 hours later. The wild-type U1 sequence conferred a 107.8±24.2-fold and a 79.9±16.6-fold increase in activation in SKMel5 and HeLa cells, respectively (Fig. 2B), confirming the ubiquitous enhancer activity of this element [19], [23]. Under our experimental conditions, the identified variation did not significantly alter U1 enhancer activity. Indeed, a 94.8±22.1-fold and an 86.1±8.8-fold increase in activation were observed in SKMel5 and HeLa cells, respectively. Altogether, our results argued that the variation identified in this patient did not confer any significant functional consequences.

### Analysis of *MITF* regulatory sequences

**Figure 3:**
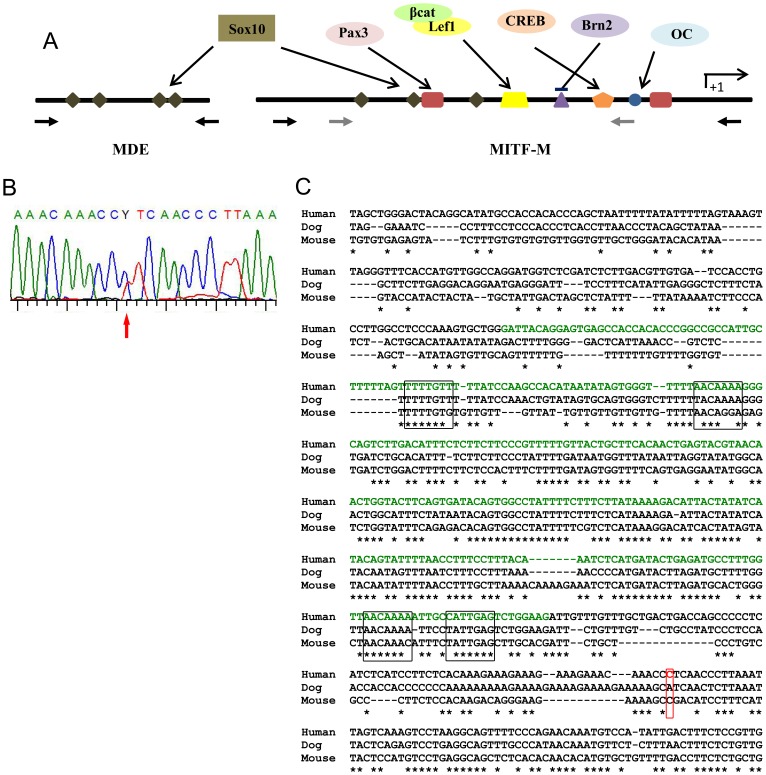
Variations identified in *MITF* regulatory regions. (A) Schematic view of the MITF-M promoter and MDE enhancer regions showing binding sites for transcription factors known to regulate *MITF/Mitf* expression in melanocytes. Note the presence of several SOX10 binding sites in both promoter and enhancer regions. Grey arrows indicate the position of QMF-PCR primers. Black arrows indicate the position of primers used for PCR and sequencing screening. (B) Electropherogram showing the heterozygous variation identified. (C) Alignment of the nucleotide sequences of human MDE (GenBank accession number NT_022459) and its corresponding *Mus musculus* (NT_039353) and canine (AC191512.6) homologous regions. The asterisks indicate the identical nucleotides between murine, canine, and human sequences. The four putative SOX10 binding sites are indicated by black boxes. The previously described human MDE 298 bp region [13] is indicated in green. The location of the identified variation is indicated by a red open box. Note that it affects a nucleotide conserved between humans and mice, but not dogs.

We used similar strategies to search for deletions and point mutations within the known *MITF* regulatory sequences. First, we used QMF-PCR to screen for deletions within the well known MITF-M promoter region (Fig. 3A, grey arrows indicate the positions of the primers). No deletion or rearrangement were identified. In parallel, we used direct PCR sequencing strategies to analyse i) the 400 bp promoter region and 100 bp downstream of the M transcription initiation site (Fig. 3A and Table 1); and ii) the MDE region and around 150 bp of flanking regions (Fig. 3A, black arrows, and Table 1 for primer sequences). No variation was identified within the promoter region, but one nucleotidic substitution, not reported in polymorphism databases, was found within the 3′ flanking region of the previously defined MDE region: g.69972010C>T on chromosome 3 (Fig. 3B). The affected nucleotide is conserved in mouse but not in dog (Fig. 3C). This variation was identified in a WS2 patient who is the unique child of a non consanguineous couple. He presented with bilateral profound sensorineural hearing impairment diagnosed at 8 months of age. The temporal bones CT scan and fundus oculi were normal. At 16 months, he presented with a synophrys without any other dymorphism. His mother was born with a white frontal forelock and her hair has begun greying at 16 years. Several cases of premature hair greying have been noted in the maternal lineage. The molecular result was confirmed on a second sample but the parents were not available for testing.

**Figure 4:**
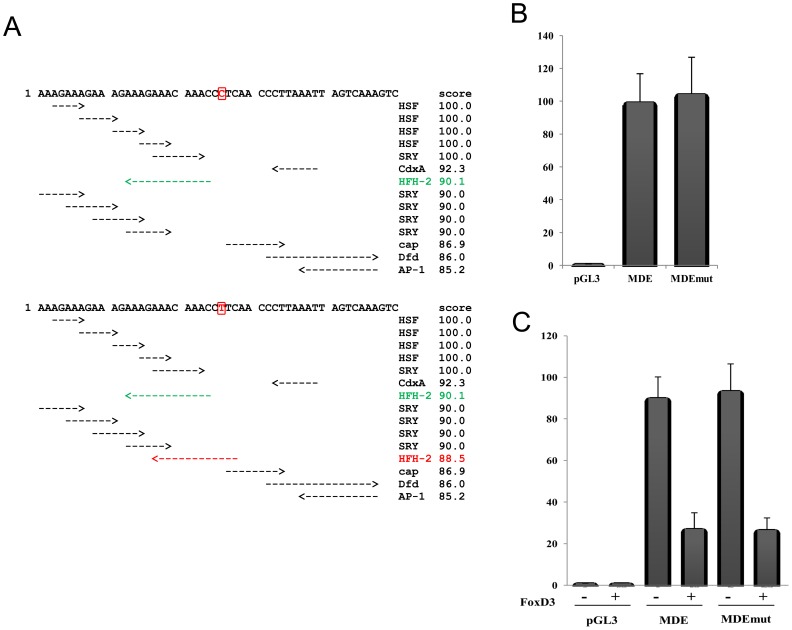
Functional consequences of the variation identified within MDE. (A) TFSEARCH results obtained upon analysis of 50 bp around the variation. The top panel corresponds to the wild-type sequence, whereas the bottom panel corresponds to the mutated sequence. The HFH2 additional binding site is indicated in red; the one present in both wild-type and mutated sequences is indicated in green. (B) Wild-type and mutated versions of the MDE reporter construct were assayed for enhancer activity in SKMel5 melanoma cell lines. Reporter gene activation is presented as fold-induction relative to the empty vector. Results represent the mean ± standard error of seven different experiments, each performed in duplicate. (C) Wild-type and mutated versions of the MDE reporter construct were co-transfected with a FoxD3 expression vector. Reporter gene activation is presented as the fold-induction relative to the empty vector. Results represent the mean ± standard error of three different experiments, each performed in duplicate.

The region containing the identified variation was described as able to reduce the enhancer activity of MDE, but no transcription factor binding sites were reported [13]. TFSEARCH analysis revealed that the region contained a putative HFH-2 (HNF-3 Forkhead homolog 2; FoxD3) binding site. The variation lies within a CAP (cAMP receptor protein) binding site and could create an additional FoxD3 putative binding site (Fig. 4A).

To test its functional relevance, the consequences of this variation on the ability of MDE to direct reporter gene expression was analysed in melanoma cells as previously described [13]. Briefly, wild-type or mutated versions of MDE (MDE or MDEmut) constructs were transfected into SKMel5 cells and their enhancer activity was tested 24 hours later. A 99.4±17.4-fold and a 104.3±22.4-fold increase in activation was observed in the wild-type and mutated versions of MDE, respectively (Fig. 4B). The presence of putative FoxD3 binding sites within the wild-type MDE sequence as well as the presence of an additional site within the patient's sequence led us to test the role of FoxD3 on MDE regulation. Co-transfection with FoxD3 reduced the fold-activation to 27±7.8, suggesting a repressive role for this transcription factor on MITF promoter activity [27], [28], [29] and on MDE (Fig. 4C). Nevertheless, no further repression was observed upon transfection with the mutated construct. Indeed, the same repressive effect was observed for the mutated MDE sequence (Fig. 4C), arguing against any pathogenic effect caused by the variation.

## Discussion

In this study, we report the screening of *SOX10* and *MITF* regulatory elements in WS2 patients that had not been previously characterised at the molecular level. No deletion was identified upon QMF-PCR analysis of the *MITF* promoter and the *SOX10* regulatory regions. Sequencing of U1 and U3 SOX10 enhancers, as well as the *MITF* promoter and enhancer regions, led to the identification of two new variations: one in close proximity to the *MITF* enhancer sequence MDE, and one within the U1 enhancer. Each of these variations could potentially create or alter transcription factor binding sites: creation of an HFH2/FOXD3 site in the case of MDE, and alteration of ADR1 and AP2 binding sites in the case of U1. However, combined functional analyses and familial segregation suggested an absence of deleterious effects for these two variations.

For MITF, TFSEARCH analysis revealed that the 298 bp MDE region contained at least two putative HFH2/FOXD3 binding sites. One of these, described in Figure 4, is very close to the variation identified. Interestingly, recent reports shed light on the involvement of FOXD3 in the regulation of *Mitf* expression *in vivo* and *in vitro*. Several groups working in mouse or zebrafish showed that this transcription factor represses *MITF* expression through promoter regulation [28], [29], [30], [31]. However, direct binding of FOXD3 to the *MITF* promoter region has been proposed by some and refuted by others. Thomas *et al.* suggested that functional FoxD3 binding sites might exist elsewhere in the gene [31]. Our results are in agreement with this last observation and suggest that FoxD3 also represses *MITF* expression through MDE regulation.

Recent reports have found that the disruption of highly conserved non-coding elements, both within or at a long distance from the coding sequences of key genes, resulted in several neurocristopathies, including HD and WS type 4 ([24], [26]). These findings serve to open new routes to the molecular description of these disorders. The *MITF* and *SOX10* regulatory sequences were therefore considered to be good candidates for yet unexplained WS2 cases. The very low level of sequence variation we identified here argues against a major implication of these regulatory sequences in WS2 and leaves about 70% of WS2 still unexplained at the molecular level. Future studies will aim at screening the noncoding regions of these genes, but priority should be given to the discovery of new WS2 genes.

The recent identification of a deletion encompassing the *SOX10* regulatory elements U1 and U3 in a patient with WS4 [24], as well as their functional importance during enteric nervous system development ([22] and our unpublished observations), opens the possibility that variations within regulatory sequences could be at the origin of other phenotypes, or play a role in phenotypic variability. This paradigm parallels another *SOX* gene close to *SOX10*, *SOX9*. The involvement of the latter in campomelic dysplasia (CD) was demonstrated 17 years ago [32], [33], but long distance genomic alterations at this locus have been recently associated with isolated disorders of sex development as well as isolated Pierre Robin sequence (PRS), both typical features of CD [34], [35], [36], [37]. In addition to enlarging the list of diseases associated with *SOX9* mutations in human, these results clearly demonstrate that endophenotypes of CD can result from tissue-restricted alterations of *SOX9* expression due to disruption of tissue-specific, long-distance regulatory regions [26], [37]. The increasing number of *SOX10* regulatory elements identified and the tissue-specific expression patterns of some of them lead us to speculate that endophenotypes of WS4, such as isolated HD, might also be linked to mutations within regulatory sequences of *SOX10*, hypothesis that will be tested in the near future.

## References

[1] Pingault V, Ente D, Dastot-Le Moal F, Goossens M, Marlin S, et al (2010) Review and update of mutations causing Waardenburg syndrome. Hum Mutat 31: 391–406. 20127975 10.1002/humu.21211

[2] Read AP, Newton VE (1997) Waardenburg syndrome. J Med Genet 34: 656–665. 9279758 10.1136/jmg.34.8.656 PMC1051028

[3] Sanchez-Martin M, Rodriguez-Garcia A, Perez-Losada J, Sagrera A, Read AP, et al (2002) SLUG (SNAI2) deletions in patients with Waardenburg disease. Hum Mol Genet 11: 3231–3236. 12444107 10.1093/hmg/11.25.3231

[4] Bondurand N, Dastot-Le Moal F, Stanchina L, Collot N, Baral V, et al (2007) Deletions at the SOX10 gene locus cause Waardenburg syndrome types 2 and 4. Am J Hum Genet 81: 1169–1185. 17999358 10.1086/522090 PMC2276340

[5] Chaoui A, Watanabe Y, Touraine R, Baral V, Goossens M, et al (2011) Identification and functional analysis of SOX10 missense mutations in different subtypes of waardenburg syndrome. Hum Mutat 32: 1436–1449. 21898658 10.1002/humu.21583

[6] Inoue K, Khajavi M, Ohyama T, Hirabayashi S, Wilson J, et al (2004) Molecular mechanism for distinct neurological phenotypes conveyed by allelic truncating mutations. Nat Genet 36: 361–369. 15004559 10.1038/ng1322

[7] Arnheiter H (2011) The discovery of the microphthalmia locus and its gene, Mitf. Pigment Cell Melanoma Res 23: 729–735. 10.1111/j.1755-148X.2010.00759.x PMC2964399 20807369

[8] Shibahara S, Takeda K, Yasumoto K, Udono T, Watanabe K, et al (2001) Microphthalmia-associated transcription factor (MITF): multiplicity in structure, function, and regulation. J Investig Dermatol Symp Proc 6: 99–104. 10.1046/j.0022-202x.2001.00010.x 11764295

[9] Steingrimsson E, Copeland NG, Jenkins NA (2004) Melanocytes and the microphthalmia transcription factor network. Annu Rev Genet 38: 365–411. 15568981 10.1146/annurev.genet.38.072902.092717

[10] Hou L, Pavan WJ (2008) Transcriptional and signaling regulation in neural crest stem cell-derived melanocyte development: do all roads lead to Mitf? Cell Res 18: 1163–1176. 19002157 10.1038/cr.2008.303

[11] Sommer L (2010) Generation of melanocytes from neural crest cells. Pigment Cell Melanoma Res 24: 411–421. 10.1111/j.1755-148X.2011.00834.x 21310010

[12] Vance KW, Goding CR (2004) The transcription network regulating melanocyte development and melanoma. Pigment Cell Res 17: 318–325. 15250933 10.1111/j.1600-0749.2004.00164.x

[13] Watanabe K, Takeda K, Yasumoto K, Udono T, Saito H, et al (2002) Identification of a distal enhancer for the melanocyte-specific promoter of the MITF gene. Pigment Cell Res 15: 201–211. 12028584 10.1034/j.1600-0749.2002.01080.x

[14] Tsuchida S, Takizawa T, Abe K, Okamoto M, Tagawa M (2009) Identification of microphthalmia-associated transcription factor isoforms in dogs. Vet J 182: 283–293. 18701327 10.1016/j.tvjl.2008.06.004

[15] Harris ML, Baxter LL, Loftus SK, Pavan WJ (2010) Sox proteins in melanocyte development and melanoma. Pigment Cell Melanoma Res 23: 496–513. 20444197 10.1111/j.1755-148X.2010.00711.x PMC2906668

[16] Mollaaghababa R, Pavan WJ (2003) The importance of having your SOX on: role of SOX10 in the development of neural crest-derived melanocytes and glia. Oncogene 22: 3024–3034. 12789277 10.1038/sj.onc.1206442

[17] Wegner M (2005) Secrets to a healthy Sox life: lessons for melanocytes. Pigment Cell Res 18: 74–85. 15760336 10.1111/j.1600-0749.2005.00218.x

[18] Antonellis A, Bennett WR, Menheniott TR, Prasad AB, Lee-Lin SQ, et al (2006) Deletion of long-range sequences at Sox10 compromises developmental expression in a mouse model of Waardenburg-Shah (WS4) syndrome. Hum Mol Genet 15: 259–271. 16330480 10.1093/hmg/ddi442

[19] Antonellis A, Huynh JL, Lee-Lin SQ, Vinton RM, Renaud G, et al (2008) Identification of neural crest and glial enhancers at the mouse Sox10 locus through transgenesis in zebrafish. PLoS Genet 4: e1000174. 18773071 10.1371/journal.pgen.1000174 PMC2518861

[20] Betancur P, Bronner-Fraser M, Sauka-Spengler T (2010) Genomic code for Sox10 activation reveals a key regulatory enhancer for cranial neural crest. Proc Natl Acad Sci U S A 107: 3570–3575. 20139305 10.1073/pnas.0906596107 PMC2840498

[21] Wahlbuhl M, Reiprich S, Vogl MR, Bosl MR, Wegner M (2011) Transcription factor Sox10 orchestrates activity of a neural crest-specific enhancer in the vicinity of its gene. Nucleic Acids Res 10.1093/nar/gkr734 PMC3245941 21908409

[22] Werner T, Hammer A, Wahlbuhl M, Bosl MR, Wegner M (2007) Multiple conserved regulatory elements with overlapping functions determine Sox10 expression in mouse embryogenesis. Nucleic Acids Res 35: 6526–6538. 17897962 10.1093/nar/gkm727 PMC2095789

[23] Yokota Y, Saito D, Tadokoro R, Takahashi Y (2011) Genomically integrated transgenes are stably and conditionally expressed in neural crest cell-specific lineages. Dev Biol 353: 382–395. 21310145 10.1016/j.ydbio.2011.02.001

[24] Bondurand N, Fouquet V, Baral V, Lecerf L, Loundon N, et al (2012) Alu-mediated deletion of SOX10 regulatory elements in Waardenburg syndrome type 4. Euro J Human Genetics In press 10.1038/ejhg.2012.29 PMC3421117 22378281

[25] Amiel J, Sproat-Emison E, Garcia-Barcelo M, Lantieri F, Burzynski G, et al (2008) Hirschsprung disease, associated syndromes and genetics: a review. J Med Genet 45: 1–14. 17965226 10.1136/jmg.2007.053959

[26] Amiel J, Benko S, Gordon CT, Lyonnet S (2010) Disruption of long-distance highly conserved noncoding elements in neurocristopathies. Ann N Y Acad Sci 1214: 34–46. 21175683 10.1111/j.1749-6632.2010.05878.x

[27] Bondurand N, Pingault V, Goerich DE, Lemort N, Sock E, et al (2000) Interaction among SOX10, PAX3 and MITF, three genes altered in Waardenburg syndrome. Hum Mol Genet 9: 1907–1917. 10942418 10.1093/hmg/9.13.1907

[28] Curran K, Lister JA, Kunkel GR, Prendergast A, Parichy DM, et al (2010) Interplay between Foxd3 and Mitf regulates cell fate plasticity in the zebrafish neural crest. Dev Biol 344: 107–118. 20460180 10.1016/j.ydbio.2010.04.023 PMC2909359

[29] Curran K, Raible DW, Lister JA (2009) Foxd3 controls melanophore specification in the zebrafish neural crest by regulation of Mitf. Dev Biol 332: 408–417. 19527705 10.1016/j.ydbio.2009.06.010 PMC2716409

[30] Ignatius MS, Moose HE, El-Hodiri HM, Henion PD (2008) colgate/hdac1 Repression of foxd3 expression is required to permit mitfa-dependent melanogenesis. Dev Biol 313: 568–583. 18068699 10.1016/j.ydbio.2007.10.045 PMC2700343

[31] Thomas AJ, Erickson CA (2009) FOXD3 regulates the lineage switch between neural crest-derived glial cells and pigment cells by repressing MITF through a non-canonical mechanism. Development 136: 1849–1858. 19403660 10.1242/dev.031989 PMC2680109

[32] Foster JW, Dominguez-Steglich MA, Guioli S, Kwok C, Weller PA, et al (1994) Campomelic dysplasia and autosomal sex reversal caused by mutations in an SRY-related gene. Nature 372: 525–530. 7990924 10.1038/372525a0

[33] Wagner T, Wirth J, Meyer J, Zabel B, Held M, et al (1994) Autosomal sex reversal and campomelic dysplasia are caused by mutations in and around the SRY-related gene SOX9. Cell 79: 1111–1120. 8001137 10.1016/0092-8674(94)90041-8

[34] Benko S, Fantes JA, Amiel J, Kleinjan DJ, Thomas S, et al (2009) Highly conserved non-coding elements on either side of SOX9 associated with Pierre Robin sequence. Nat Genet 41: 359–364. 19234473 10.1038/ng.329

[35] Benko S, Gordon CT, Mallet D, Sreenivasan R, Thauvin-Robinet C, et al (2011) Disruption of a long distance regulatory region upstream of SOX9 in isolated disorders of sex development. J Med Genet 48: 825–830. 22051515 10.1136/jmedgenet-2011-100255

[36] Georg I, Bagheri-Fam S, Knower KC, Wieacker P, Scherer G, et al (2010) Mutations of the SRY-responsive enhancer of SOX9 are uncommon in XY gonadal dysgenesis. Sex Dev 4: 321–325. 20838034 10.1159/000320142

[37] Gordon CT, Tan TY, Benko S, Fitzpatrick D, Lyonnet S, et al (2009) Long-range regulation at the SOX9 locus in development and disease. J Med Genet 46: 649–656. 19473998 10.1136/jmg.2009.068361

